# The *SRGAP2* SNPs, their haplotypes and G × E interactions on serum lipid traits

**DOI:** 10.1038/s41598-017-10950-6

**Published:** 2017-09-14

**Authors:** Liu Miao, Rui-Xing Yin, Jin-Zhen Wu, Shuo Yang, Wei-Xiong Lin, Shang-Ling Pan

**Affiliations:** 1 Department of Cardiology, Institute of Cardiovascular Diseases, The First Affiliated Hospital, Guangxi Medical University, Nanning, 530021 Guangxi People’s Republic of China; 2 Department of Molecular Genetics, Medical Scientific Research Center, Guangxi Medical University, Nanning, 530021 Guangxi People’s Republic of China; 3 Department of Pathophysiology, School of Premedical Science, Guangxi Medical University, Nanning, 530021 Guangxi People’s Republic of China

## Abstract

Maonan nationality is a relatively conservative and isolated minority in China. Little is known about the association of the Slit-Robo Rho GTPase activating protein 2 gene (*SRGAP*2) single nucleotide polymorphisms (SNPs) and serum lipid levels in the Chinese populations. This study was performed to clarify the association of the *SRGAP*2 rs2483058 and rs2580520 SNPs and their haplotypes with serum lipid traits in the Maonan and Han populations. Genotyping of the 2 SNPs was performed in 2444 unrelated subjects (Han, 1210 and Maonan, 1234) by polymerase chain reaction and restriction fragment length polymorphism combined with gel electrophoresis, and then confirmed by direct sequencing. The allelic (rs2483058) and genotypic (rs2483058 and rs2580520) frequencies were different between the two ethnic groups. Four haplotypes were identified in our populations, and the rs2483058G-rs2580520C haplotype was the commonest one. The rs2483058C-rs2580520G haplotype was associated with an increased risk of dyslipidemia, and showed consistent association with serum total cholesterol (TC), high-density lipoprotein cholesterol (HDL-C), apolipoprotein (Apo) A1 levels, and the ApoA1/ApoB ratio. These results indicated that the *SRGAP*2 SNPs and their haplotypes were associated with serum lipid levels. Their haplotypes can explain much more serum lipid variation than any single SNP alone, especially for serum TC, HDL-C and ApoA1 levels.

## Introduction

During the past decades, cardiovascular disease (CVD) has become the leading cause which can give rise to the world’s largest mortality, morbidity, disability, functional decline, and healthcare costs^1, 2^. To evaluate risk severity, we general survey a standard lipid profile, just as total cholesterol (TC)^3^, triglyceride (TG)^4^, low-density lipoprotein cholesterol (LDL-C)^5^, apolipoprotein (Apo) B^6^, high-density lipoproteins cholesterol (HDL-C)^7, 8^, ApoA1^8^ and the ratio ApoA1 to ApoB^9^, which is an efficient way for cardiovascular risk prediction and can be recommended from an integral component of approaches. Several researches in the past years about CVD risk factors have showed that the morbidity was usually different between men and women^10^, was also affected by age^11^ and ethnicity^12^, and was modified by behavioral choices^13^, poor diet^14^ and unhealthy lifestyle^15^, environmental factors^16^, and personal genetic profile^17, 18^. All these risk factors which have been taken to individual are important genetic components, however, there are lots of the true magnitude risk factor are uncertain to cluster, as well as on the role of genetic factors in risk factor clustering for individuals. In that case, the target of genome-wide association studies (GWASes) was to find out which part can identify common single nucleotide polymorphisms (SNPs) and calculate the numbers of the phenotypic variance is actually located by them^19^.

Some GWASes have demonstrated that several SNPs near the SLIT-ROBO Rho GTPase activating protein 2 gene (*SRGAP*2; also knows as: *FNBP*2; *SRGAP3*; *SRGAP*2*A*; *ARHGAP34*, Gene ID: 23380, HGNC ID: 19751, synonyms: FLJ33003, FLJ42565, KIAA0456, locus type: gene with protein product, chromosomal location: 1q32.1) may result in negatively regulate neuronal migration and induce neurite outgrowth^20^. In addition, it might also contribute to the higher susceptibility to neurodegenerative or psychiatric disorders of the human brain^21^. At the same time, a large number of surveys have showed that *SRGAP2* expression was up regulated in multiple breast cancer cells. The mechanism was supposed to have a connection with lipid metabolism^22^. A previous GWAS on plasma lipid levels has identified the rs2483058 SNP near the *SRGAP2* as hyperlipidemic locus in European^23^. In the meantime, several previous studies have showed that the association between the *SRGAP2* rs2483058 SNP and serum lipid levels might have ethnic- and/or sex-specificity^24–26^. Besides these, another rs2580520 SNP has been clarified to contribute to the development of breast cancer^27^. As we have known that the hyperlipidemia may be an important part to result in breast cancer^28^, whether the *SRGAP2* rs2483058 and rs2580520 SNPs are associated with serum lipid levels or whether they show ethnic- and/or sex-specific association as the previous reports remains dubious.

As we all know that China is a multi-ethnic country, including 56 nationalities. Han is the largest group and Maonan is one of the 55 minorities with a population of 107,166 (Rank 37) according to the sixth national census statistics of China in 2010. The Maonan people are mainly distributed in the Shangnan, Zhongnan, and Xianan townships of Huanjiang Maonan Autonomous County in the north of the Guangxi Zhuang Autonomous Region, which is situated in Southwestern China. Several previous studies have showed that the genetic relationship between Maonan nationality and other minorities in Guangxi^29^ was much closer than that between Maonan and Han nationalities^30^. In spite of a very small population, the Maonan ethnic group is well known in China for its long history and unique culture. The special customs and culture, including their clothing, intra-ethnic marriages, dietary habits and lifestyle factors are different from those of local Han Chinese^31^. They have their culture of consanguineous marriage to cousins of maternal side, suggesting that the genetic background of Maonan population may be less heterogeneous within the population. This study, therefore, was undertaken to detect the association of the *SRGAP2* rs2483058 and rs2580520 SNPs and several environmental factors with serum lipid levels between males and females in the Maonan and Han populations.

## Results

### Demographic and biochemical characteristics

The demographic and biochemical characteristics of the participants according to ethnic group are presented in Table 1. The levels of body weight, body mass index (BMI), waist circumference, systolic blood pressure, diastolic blood pressure, pulse pressure and blood glucose and the percentages of subjects who consumed alcohol were higher in Maonan than in Han (*P* < 0.05- *P* < 0.001), whereas the levels of body height and the percentages of subjects who smoked cigarettes were lower in Maonan than in Han (*P* < 0.05). There was no significant difference in the levels of age, sex ratio, TC, TG, LDL-C, HDL-C, ApoA1, ApoB and the ratio of ApoA1 to ApoB (*P* > 0.05 for all).

**Table 1:** Comparison of demographic, lifestyle characteristics and serum lipid levels between the Han and Maonan populations.

Parameter	Han	Maonan	*t* (*x* ^*2*^)	*P*
Number	1210	1234		
Male/female	465/745	500/734	1.116	0.291
Age (years)^1^	55.77 ± 13.89	56.31 ± 13.80	2.944	0.086
Height (cm)	153.95 ± 7.70	153.80 ± 8.12	5.470	0.019
Weight (kg)	52.76 ± 8.75	53.30 ± 10.81	38.733	1.9E-009
Body mass index (kg/m^2^)	22.42 ± 3.30	22.43 ± 3.74	5.104	0.024
Waist circumference	74.94 ± 7.79	76.87 ± 9.15	25.601	1.8E-007
Smoking status [*n*(%)]				
Non-smoker	902 (74.55)	969 (78.53)		
≤20 cigarettes/day	207 (17.11)	199 (16.13)		
>20 cigarettes/day	101 (8.34)	66 (5.34)	9.657	0.008
Alcohol consumption [*n*(%)]				
Non-drinker	971 (80.25)	975 (79.01)		
≤25 g/day	101 (8.35)	146 (11.83)		
>25 g/day	138 (11.40)	113 (9.16)	10.462	0.005
Systolic blood pressure (mmHg)	128.59 ± 19.77	136.14 ± 23.30	29.768	2.1E-007
Diastolic blood pressure (mmHg)	80.80 ± 10.92	83.21 ± 11.84	6.869	0.009
Pulse pressure (mmHg)	47.79 ± 14.87	52.94 ± 17.23	25.919	1.9E-007
Glucose (mmol/L)	6.15 ± 1.77	6.20 ± 1.40	21.853	1.2E-006
Total cholesterol (mmol/L)	4.80 ± 1.08	5.23 ± 1.04	0.003	0.958
Triglyceride (mmol/L)^2^	1.43 (0.66)	1.60 (0.72)	0.455	0.327
HDL-C (mmol/L)	2.04 ± 0.52	1.51 ± 0.39	0.823	0.364
LDL-C (mmol/L)	2.83 ± 0.83	2.82 ± 0.79	0.442	0.506
Apolipoprotein (Apo) A1 (g/L)	1.27 ± 0.17	1.28 ± 0.19	0.006	0.941
ApoB (g/L)	0.84 ± 0.20	0.88 ± 0.19	0.609	0.435
ApoA1/ApoB	1.59 ± 0.41	1.53 ± 0.42	0.193	0.660

*HDL-C*, high-density lipoprotein cholesterol; *LDL-C*, low-density lipoprotein cholesterol. ^1^The quantitative variables were presented as mean ± standard deviation and determined by Student’s unpaired t-test. ^2^The value of triglyceride was presented as median (interquartile range) for not meet the normal distribution, the difference between the two ethnic groups was determined by the Wilcoxon-Mann-Whitney test.

### Results of electrophoresis and genotyping

**Figure 1:**
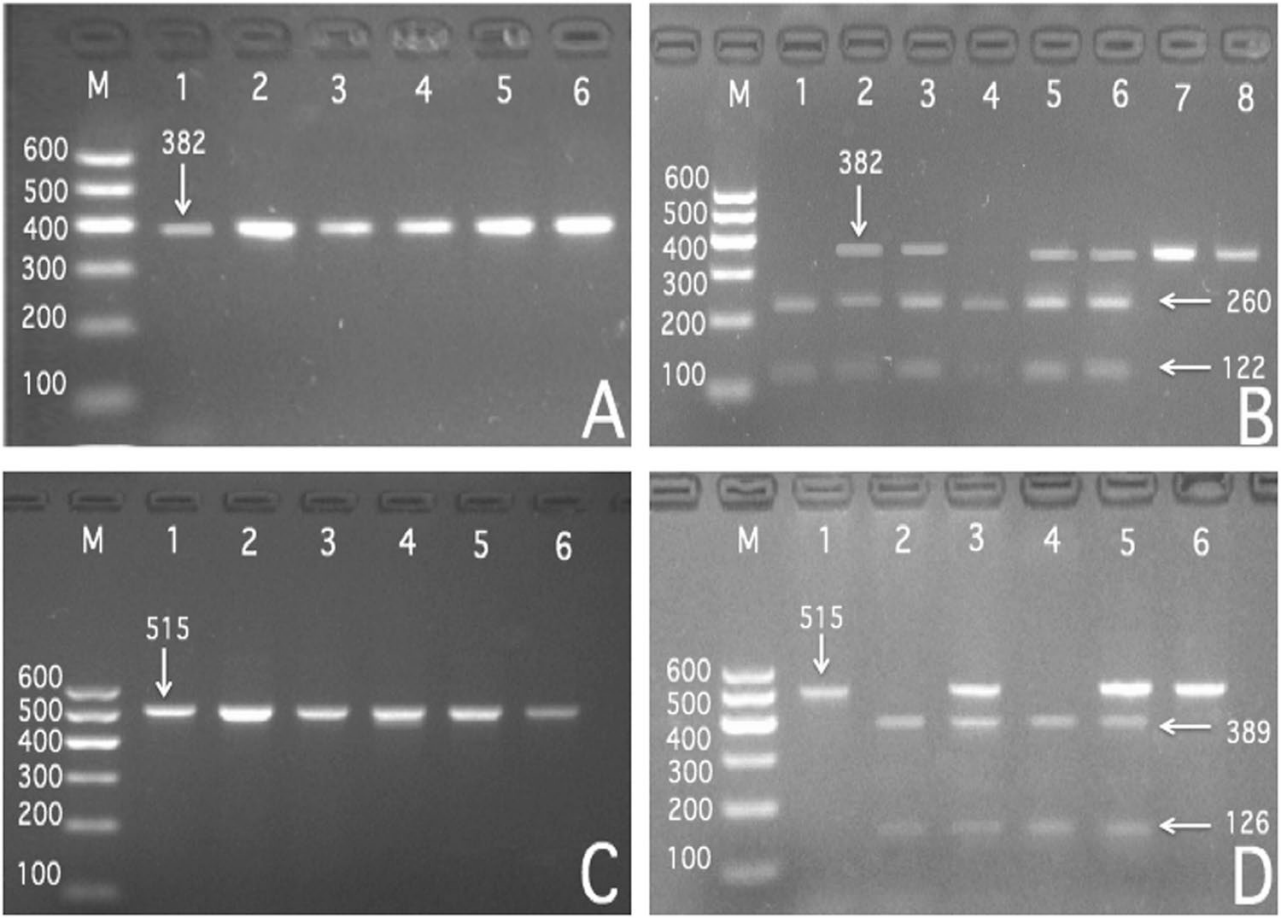
Agarose gel electrophoresis (2%) of PCR products and genotyping of the *SRGAP2* SNPs. (**A**) and (**B**) (rs2483058): Lane M, 100 bp marker ladder; lanes A1-A6, 382 bp band samples; lanes B1 and B4, CC genotype (260- and 122-bp); lanes B2, B3, B5 and B6, GC genotype (382-, 260- and 122-bp); Lanes B7 and B8, GG (382-bp). (**C**) and (**D)** (rs2580520): Lane M, 100 bp marker ladder; lanes C1-C6, 515 bp band samples; lanes D1 and D6, GG genotype (515-bp); lanes D3 and D5, GC genotype (515-, 389- and 126-bp); lanes D2 and D4, CC (389- and 126-bp).

**Figure 2:**
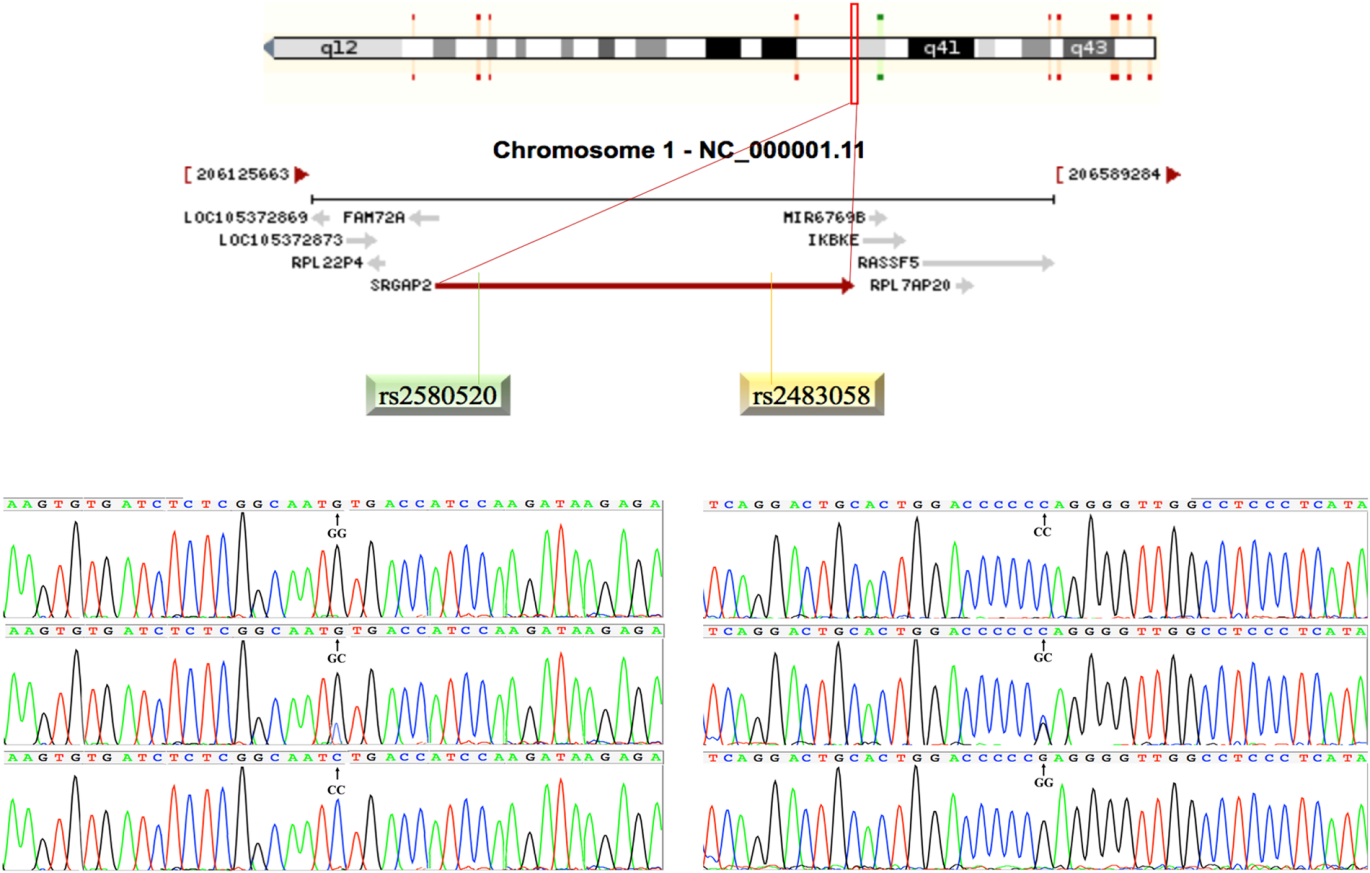
The positions of the *SRGAP2* rs2580520 and rs2483058 variants.

After the genomic DNA of the samples was amplified by polymerase chain reaction (PCR) and imaged by agarose gel electrophoresis for the *SRGAP2* rs2483058 SNP, the PCR product of 382-bp nucleotide sequences could be seen in the samples (Figs 1 and 2). The GG (382 bp), GC (382-, 260- and 122-bp) and CC (260- and 122-bp) genotypes were shown, respectively. The PCR product of the rs2580520 SNP was 515-bp nucleotide sequences. The GG (515 bp), GC (515-, 389-, and 126-bp) and CC (389- and 126- bp) genotypes were shown, respectively.

### Genotypic and allelic frequencies

The genotypic distribution of the two loci was in Hardy-Weinberg equilibrium (*P* > 0.05 for all). The genotypic and allelic frequencies of the rs2483058 SNP were different between Maonan and Han, the frequencies of the rs2483058C allele (41.86% *vs*. 37.07%, *P* = 0.001) and rs2483058GC genotype (48.06% *vs*. 40.90%, *P* < 0.001) were higher in Maonan than in Han. Subgroup analysis showed that the rs2483058C allele frequency was higher in Han females than in Han males (39.06% *vs*. 33.87%, *P* = 0.01), but it was lower in Maonan females than in Maonan males (39.37% *vs*. 45.50%, *P* = 0.002). The genotypic frequencies of the rs2580520 SNP between Maonan and Han, and between Han males and Han females were also different (*P* < 0.05-*P* < 0.005; Table 2).

**Table 2:** Comparison of the genotype and allele frequencies of the *SRGAP2* SNPs in the Han and Maonan populations [*n* (%)]. *HWE*, Hardy-Weinberg equilibrium. n = sample size.

Group	*n*	Genotype			Allele	
rs2483058		GG	GC	CC	G	C
Han	1210	514 (42.48)	495 (40.90)	201 (16.62)	1523 (62.93)	897 (37.07)
Maonan	1234	421 (34.12)	593 (48.06)	220 (17.82)	1435 (58.14)	1033 (41.86)
*x* ^*2*^		18.701			11.731	
*P*		9E-005			0.001	
*P*_HWE_			0.357			
Han						
Male	465	216(46.45)	183(39.35)	66(14.20)	615(66.13)	315(33.87)
Female	745	298(40.00)	312(41.88)	135(18.12)	908(60.94)	582(39.06)
*x* ^*2*^		5.910			6.610	
*P*		0.052			0.010	
*P*_HWE_			0.423			
Maonan						
Male	500	151(30.20)	243(48.60)	106(21.20)	545(54.50)	455(45.50)
Female	734	270(36.78)	350(47.68)	114(15.54)	890(60.63)	578(39.37)
*x* ^*2*^		9.192			9.174	
*P*			0.010		0.002	
*P*_HWE_		0.566				
rs2580520						
Han	1210	89(7.36)	419(34.64)	702(58.01)	597(24.67)	1823(75.33)
Maonan	1234	73(5.91)	509(41.25)	652(52.84)	655(26.54)	1813(73.46)
*x* ^*2*^		11.921			2.243	
*P*		0.003			0.134	
*P*_HWE_			0.529			
Han						
Male	465	23(4.94)	158(33.98)	284(61.08)	204(21.94)	726(78.06)
Female	745	66(8.86)	261(35.03)	418(56.11)	393(26.38)	1097(73.62)
*x* ^*2*^		7.269			6.075	
*P*		0.026			0.014	
*P*_HWE_			0.448			
Maonan						
Male	500	26(5.20)	211(42.20)	263(52.60)	263(26.30)	737(73.70)
Female	734	47(6.41)	298(40.60)	389(52.99)	392(26.70)	1076(73.30)
*x* ^*2*^		0.921			0.050	
*P*		0.631			0.853	
*P*_HWE_		0.772				

### Genotypes and serum lipid levels

Tables 3 and 4 describe the association between genotypes and serum lipid levels. The levels of ApoA1 and the ratio of ApoA1 to ApoB in both Han and Maonan were different among the rs2483058 genotypes (*P* < 0.01 for all), the rs2483058C allele carriers had lower ApoA1 levels and the ApoA1/ApoB ratio than the rs2483058C allele non-carriers. The concentration of HDL-C in Maonan but not in Han was also different among the rs2483058 genotypes (*P* = 0.024), the rs2483058C allele carriers had lower HDL-C concentration than the rs2483058C allele non-carriers. The levels of TC, LDL-C and ApoB in Maonan but not in Han were different among the rs2580520 genotypes (*P* = 0.011-*P* = 0.001), the rs2580520C allele carriers had higher TC, LDL-C and ApoB levels than the rs2580520C allele non-carriers. Subgroup analysis showed that the levels of ApoA1 and the ratio of ApoA1 to ApoB in Han males and females and Maonan males were different among the rs2483058 genotypes (*P* < 0.01 for all), the rs2483058C allele carriers had lower ApoA1 levels and the ApoA1/ApoB ratio than the rs2483058C allele non-carriers. The levels of ApoA1 in Maonan females were also different among the rs2483058 genotypes (*P* < 0.001), the rs2483058C allele carriers had lower ApoA1 levels than the rs2483058C allele non-carriers. The levels of TC in Maonan males and females were different among the rs2580520 genotypes (*P* = 0.018-*P* = 0.001), the rs2580520C allele carriers had higher TC levels than the rs2580520C allele non-carriers. The ratio of ApoA1 to ApoB in Han males and Maonan males was also different among the rs2580520 genotypes (*P* = 0.013-*P* = 0.007), the rs2580520C allele carriers had lower the ApoA1/ApoB ratio than the rs2580520C allele non-carriers.

**Table 3:** Comparison of the genotypes and serum lipid levels in the Han and Maonan populations.

Genotype	*n*	TC (mmol/L)	TG (mmol/L)	HDL-C (mmol/L)	LDL-C (mmol/L)	ApoA1 (g/L)	ApoB (g/L)	ApoA1 /ApoB
**rs2483058**								
Han								
GG	514	4.81 ± 0.93	1.41 (0.60)	2.09 ± 0.57	2.87 ± 0.73	1.29 ± 0.14	0.84 ± 0.17	1.60 ± 0.37
GC	495	4.75 ± 1.22	1.43 (0.60)	2.03 ± 0.64	2.79 ± 0.88	1.28 ± 0.17	0.83 ± 0.22	1.63 ± 0.44
CC	201	4.89 ± 1.07	1.42 (0.72)	1.99 ± 0.52	2.93 ± 0.81	1.20 ± 0.22	0.86 ± 0.20	1.47 ± 0.41
*F*		1.234	0.162	3.449	2.311	19.174	1.999	10.746
*P*		0.291	0.741	0.037	0.100	9.5E-005	0.136	1.1E-006
Maonan								
GG	421	5.23 ± 1.11	1.46 (0.77)	1.53 ± 0.39	2.81 ± 0.82	1.29 ± 0.16	0.87 ± 0.20	1.56 ± 0.45
GC	593	5.22 ± 0.98	1.44 (0.73)	1.49 ± 0.38	2.84 ± 0.76	1.30 ± 0.17	0.88 ± 0.18	1.55 ± 0.38
CC	220	5.25 ± 1.03	1.44 (0.67)	1.45 ± 0.40	2.80 ± 0.80	1.20 ± 0.19	0.89 ± 0.19	1.44 ± 0.42
*F*		0.074	0.223	3.303	0.322	24.020	0.308	7.037
*P*		0.929	0.451	0.024	0.725	1.8E-007	0.735	0.001
**rs2580520**								
Han								
GG	89	4.66 ± 1.06	1.44 (0.61)	2.04 ± 0.64	2.90 ± 0.79	1.27 ± 0.15	0.85 ± 0.20	1.57 ± 0.37
GC	419	4.69 ± 1.09	1.43 (0.63)	2.09 ± 0.54	2.80 ± 0.82	1.27 ± 0.23	0.82 ± 0.19	1.63 ± 0.46
CC	702	4.83 ± 1.14	1.42 (0.76)	1.98 ± 0.51	2.76 ± 0.94	1.26 ± 0.19	0.83 ± 0.15	1.60 ± 0.49
*F*		3.662	0.162	1.459	2.589	0.318	2.371	3.128
*P*		0.026	0.651	0.233	0.076	0.727	0.094	0.044
Maonan								
GG	73	5.17 ± 1.03	1.41 (0.62)	1.51 ± 0.38	2.79 ± 0.73	1.28 ± 0.12	0.86 ± 0.18	1.55 ± 0.41
GC	509	5.23 ± 0.99	1.43 (0.63)	1.47 ± 0.40	2.83 ± 0.83	1.27 ± 0.21	0.88 ± 0.20	1.52 ± 0.42
CC	652	5.77 ± 1.23	1.42 (0.74)	1.52 ± 0.43	3.01 ± 0.96	1.31 ± 0.39	0.93 ± 0.19	1.48 ± 0.40
*F*		11.127	0.182	1.781	4.491	2.308	4.958	1.635
*P*		1.2E-006	0.622	0.169	0.011	0.100	0.007	0.195

*TC*, total cholesterol; *TG*, triglyceride; *HDL-C*, high-density lipoprotein cholesterol; *LDL-C*, low-density lipoprotein cholesterol; *ApoA1*, apolipoprotein A1; *ApoB*, apolipoprotein B; *ApoA1/ApoB*, the ratio of apolipoprotein A1 to apolipoprotein B. The value of triglyceride was presented as median (interquartile range) for not meet the normal distribution, the difference among the genotypes was determined by the Kruskal-Wallis test. The *P*-value calculated by ANCOVA, using general linear models, and adjusted for age, sex, BMI, smoking status, alcohol use, glucose and hypertension, *P* < 0.025 was considered statistically significant (corresponding to *P* < 0.05 after adjusting for 2 independent tests by the Bonferroni correction). n = sample size.

**Table 4:** Comparison of the genotypes and serum lipid levels between males and females in the Han and Maonan populations.

Ethnic/ Genotype	*n*	TC (mmol/L)	TG (mmol/L)	HDL-C (mmol/L)	LDL-C (mmol/L)	ApoA1 (g/L)	ApoB (g/L)	ApoA1 /ApoB
**rs2483058**								
Han/male								
GG	216	4.93 ± 0.78	1.31 (0.67)	2.01 ± 0.57	2.83 ± 0.64	1.28 ± 0.16	0.86 ± 0.15	1.53 ± 0.30
GC	183	5.03 ± 1.26	1.30 (0.66)	2.04 ± 0.46	2.87 ± 0.90	1.26 ± 0.12	0.89 ± 0.24	1.57 ± 0.47
CC	66	5.04 ± 0.90	1.33 (0.84)	1.96 ± 0.39	2.91 ± 0.70	1.24 ± 0.15	0.90 ± 0.17	1.36 ± 0.35
*F*		2.877	0.603	0.475	1.183	7.479	0.508	7.421
*P*		0.238	0.546	0.622	0.345	0.001	0.402	0.001
Han/Female								
GG	298	4.79 ± 1.02	1.36 (0.66)	2.16 ± 0.56	2.91 ± 0.79	1.30 ± 0.14	0.83 ± 0.17	1.65 ± 0.41
GC	312	4.71 ± 1.16	1.35 (0.66)	2.14 ± 0.73	2.75 ± 0.83	1.26 ± 0.18	0.79 ± 0.19	1.67 ± 0.42
CC	135	4.77 ± 1.12	1.35 (0.85)	2.01 ± 0.59	2.83 ± 0.84	1.19 ± 0.25	0.82 ± 0.21	1.53 ± 0.43
*F*		2.213	0.557	3.559	2.717	17.657	2.803	5.419
*P*		0.311	0.443	0.029	0.067	8.1E-005	0.061	0.005
Maonan/male								
GG	151	5.16 ± 0.97	1.32 (0.67)	1.49 ± 0.46	2.65 ± 0.88	1.29 ± 0.20	0.86 ± 0.23	1.60 ± 0.59
GC	243	5.18 ± 0.92	1.33 (0.67)	1.42 ± 0.36	2.81 ± 0.76	1.28 ± 0.17	0.89 ± 0.18	1.50 ± 0.38
CC	106	5.28 ± 1.02	1.34 (0.88)	1.40 ± 0.44	2.79 ± 0.74	1.21 ± 0.22	0.90 ± 0.17	1.39 ± 0.37
*F*		0.529	0.224	2.662	1.929	5.683	1.273	7.174
*P*		0.590	0.821	0.043	0.146	0.004	0.281	0.001
Maonan/Female								
GG	270	5.26 ± 1.18	1.31 (0.65)	1.58 ± 0.35	2.89 ± 0.76	1.29 ± 0.26	0.88 ± 0.19	1.54 ± 0.35
GC	350	5.24 ± 1.02	1.34 (0.62)	1.52 ± 0.39	2.85 ± 0.77	1.30 ± 0.15	0.87 ± 0.18	1.58 ± 0.38
CC	114	5.22 ± 1.03	1.33 (0.77)	1.49 ± 0.36	2.81 ± 0.90	1.20 ± 0.12	0.84 ± 0.22	1.48 ± 0.45
*F*		0.054	0.904	1.897	0.456	22.151	0.305	2.877
*P*		0.947	0.216	0.029	0.634	1.3E-006	0.737	0.057
**rs2580520**								
Han/male								
GG	23	4.53 ± 0.95	1.34 (0.71)	1.92 ± 0.41	2.81 ± 0.64	1.27 ± 0.15	0.82 ± 0.23	1.73 ± 0.76
GC	158	4.86 ± 1.01	1.32 (0.69)	2.06 ± 0.55	2.82 ± 0.90	1.28 ± 0.17	0.86 ± 0.18	1.54 ± 0.39
CC	284	5.03 ± 0.99	1.33 (0.70)	1.99 ± 0.48	2.95 ± 0.70	1.29 ± 0.12	0.90 ± 0.20	1.49 ± 0.32
*F*		3.465	0.523	1.202	1.283	0.237	2.980	5.504
*P*		0.032	0.437	0.301	0.299	0.789	0.051	0.007
Han/Female								
GG	66	4.57 ± 1.17	1.32 (0.59)	1.99 ± 0.53	2.87 ± 0.96	1.26 ± 0.20	0.83 ± 0.12	1.54 ± 0.33
GC	261	4.75 ± 1.13	1.30 (0.61)	2.10 ± 0.53	2.78 ± 0.84	1.27 ± 0.21	0.79 ± 0.19	1.68 ± 0.49
CC	418	4.94 ± 1.06	1.31 (0.83)	2.08 ± 0.72	2.86 ± 0.81	1.25 ± 0.15	0.81 ± 0.20	1.61 ± 0.38
*F*		3.581	0.649	0.757	0.765	0.898	1.004	3.325
*P*		0.028	0.403	0.469	0.466	0.408	0.367	0.037
Maonan/male								
GG	26	5.08 ± 1.31	1.32 (0.67)	1.45 ± 0.42	2.79 ± 1.03	1.34 ± 0.21	0.91 ± 0.20	1.42 ± 0.34
GC	211	5.26 ± 0.95	1.33 (0.67)	1.41 ± 0.43	2.79 ± 0.85	1.25 ± 0.24	0.90 ± 0.20	1.44 ± 0.42
CC	263	5.92 ± 0.88	1.34 (0.88)	1.45 ± 0.40	2.69 ± 0.71	1.27 ± 0.15	0.89 ± 0.17	1.57 ± 0.49
*F*		10.387	0.214	0.500	0.755	2.991	2.173	4.387
*P*		1.1E-006	0.823	0.607	0.446	0.051	0.163	0.013
Maonan/Female								
GG	47	5.20 ± 1.19	1.38 (0.66)	1.56 ± 0.43	2.99 ± 0.92	1.30 ± 0.17	0.90 ± 0.19	1.56 ± 0.43
GC	298	5.23 ± 1.02	1.39 (0.61)	1.50 ± 0.37	2.86 ± 0.82	1.29 ± 0.18	0.87 ± 0.20	1.51 ± 0.41
CC	389	5.46 ± 1.11	1.35 (0.71)	1.55 ± 0.35	2.84 ± 0.73	1.27 ± 0.10	0.86 ± 0.19	1.54 ± 0.35
*F*		4.409	0.889	1.220	0.923	0.954	1.060	0.663
*P*		0.018	0.346	0.296	0.398	0.385	0.347	0.516

*TC*, total cholesterol; *TG*, triglyceride; *HDL-C*, high-density lipoprotein cholesterol; *LDL-C*, low-density lipoprotein cholesterol; *ApoA1*, apolipoprotein A1; *ApoB*, apolipoprotein B; *ApoA1/ApoB*, the ratio of apolipoprotein A1 to apolipoprotein B. The value of triglyceride was presented as median (interquartile range)for not meet the normal distribution, the difference among the genotypes was determined by the Kruskal-Wallis test.The *P*-value calculated by *ANCOVA*, using general linear models, and adjusted for age, sex, BMI, smoking status, alcohol use, glucose and hypertension, *P* < 0.025 was considered statistically significant (corresponding to *P* < 0.05 after adjusting for 2 independent tests by the Bonferroni correction). n = sample size.

### Haplotypes and serum lipid levels

**Figure 3:**
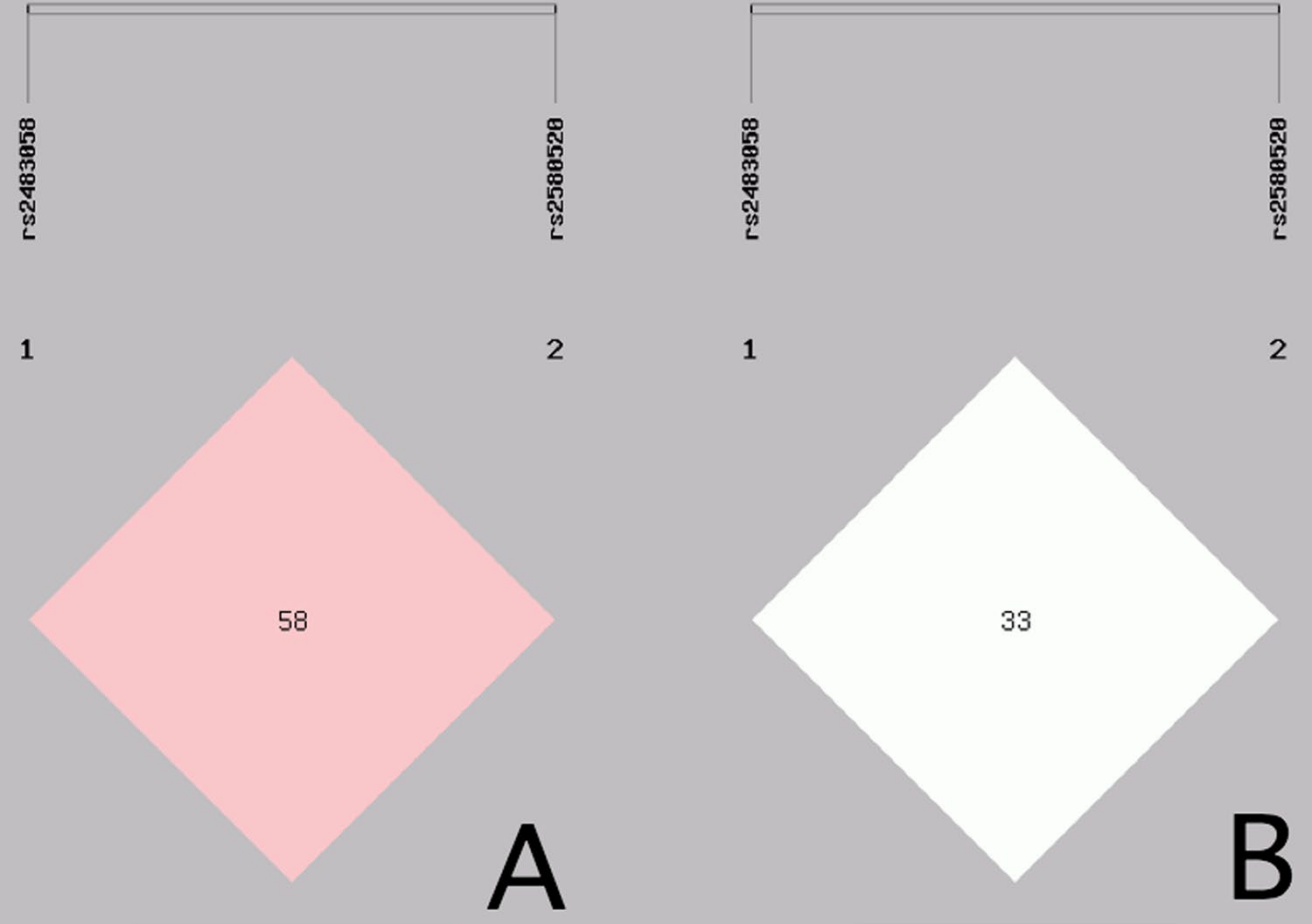
The linkage disequilibrium (LD) of the *SRGAP2* rs2483058 and rs2580520 SNPs in the combined population of Maonan and Han. The LD status is expounded by the (**A**) *D*′ = 0.58, (**B**) *r* ^2^ = 0.33.

A weak linkage disequilibrium (LD) was noted between the two SNPs (*D*′ = 0.58, *r* ^2^ = 0.33; Fig. 3). Thus, a haplotype analysis was conducted (Table 5). There were 4 haplotypes identified in our study populations. The haplotype of rs2483058G-rs2580520C was the commonest one (45%). The haplotype of rs2483058C-rs2580520G was associated with an increased risk of dyslipidemia (OR: 1.44, 95% CI: 1.17–1.78, *P* < 0.001), it showed consistent association with serum TC, HDL-C, ApoA1 and the ratio of ApoA1 to ApoB. Multivariate logistic analysis showed that the rs2483058C-rs2580520G haplotype was positively correlated with the incidence of hyperlipidemia in Han and Maonan according to stratified risk factors (gender, BMI, smoking, diabetes and blood pressure; Table 6). In addition, the rs2483058C-rs2580520G haplotype carriers had lower HDL-C and ApoA1 levels in Han and Maonan, lower ApoA1/ApoB ratio in Han, and higher TC in Maonan than the rs2483058C-rs2580520G haplotype non-carriers (Table 7).

**Table 5:** Haplotype frequencies among the 2 *SRGAP2* SNPs in the Maonan and Han populations [n (%)].

Haplotype	Total	Han	Maonan	*P*-value	OR (95%CI)
rs2483058G-rs2580520C	1102 (45)	567 (47)	540 (44)	—	—
rs2483058C-rs2580520C	716 (29)	344 (28)	370 (30)	0.086	1.14 (0.98–1.31)
rs2483058G-rs2580520G	376 (15)	193 (16)	185 (15)	0.986	1.01 (0.82–1.22)
rs2483058C-rs2580520G	250 (11)	106 (9)	148 (11)	<0.001	1.44 (1.17–1.78)

**Table 6:** The *SRGAP2* rs2483058C-rs2580520G haplotype and hyperlipidemia in the Han and Maonan populations according to stratified risk factors.

Factor	Type	Haplotype	OR (95%CI)_Han_	*P*_Han_	OR (95%CI)_Maonan_	*P*_Maonan_
Gender	Male	C-G non-carriers	1	—	1	—
	Female	C-G carriers	1.88 (1.33–2.66)	<0.001	1.24 (0.95–1.62)	<0.001
BMI	<24 Kg/m^2^	C-G non-carriers	1	—	1	—
	≥24 Kg/m^2^	C-G carriers	1.57 (1.23–2.02)	<0.001	1.26 (0.83–1.91)	0.001
Smoking	Nonsmoker	C-G non-carriers	1	—	1	—
	Smoker	C-G carriers	1.25 (0.99–1.58)	0.003	3.01 (1.77–5.11)	<0.001
Drinking	Nondrinker	C-G non-carriers	1	—	1	—
	Drinker	C-G carriers	0.88 (0.64–1.72)	0.877	1.01 (0.92–1.23)	0.654
Diabetes	Non-diabetes	C-G non-carriers	1	—	1	—
	Diabetes	C-G carriers	1.46 (1.16–1.86)	<0.001	1.53 (0.96–2.51)	0.002
Blood pressure	Normotensive	C-G non-carriers	1	—	1	—
	Hypertension	C-G carriers	1.40 (0.85–2.05)	<0.001	1.68 (1.22–2.31)	<0.001

**Table 7:** Lipid profiles according to the *SRGAP2* rs2483058C-rs2580520G haplotype in the two ethnic groups.

Group	*n*	TC (mmol/L)	TG (mmol/L)	HDL-C (mmol/L)	LDL-C (mmol/L)	ApoA1 (g/L)	ApoB (g/L)	ApoA1 /ApoB
Han plus Maonan	2444							
Carrier	250	5.32 ± 1.06	1.50 (0.69)	1.71 ± 0.58	2.86 ± 0.79	1.22 ± 0.14	0.88 ± 0.19	1.47 ± 0.47
Non-carrier	2194	5.12 ± 1.22	1.54 (0.75)	1.78 ± 0.50	2.83 ± 0.94	1.28 ± 0.17	0.86 ± 0.20	1.57 ± 0.40
*F*		11.231	0.164	3.026	2.333	33.325	2.694	6.540
*P*		0.001	0.689	0.074	0.187	8E-008	0.101	0.011
Han	1210							
Carrier	106	4.98 ± 1.08	1.42 (0.73)	1.96 ± 0.47	2.85 ± 0.81	1.20 ± 0.20	0.85 ± 0.19	1.50 ± 0.51
Non-carrier	1104	4.80 ± 1.07	1.48 (0.79)	2.06 ± 0.61	2.81 ± 0.92	1.27 ± 0.16	0.83 ± 0.20	1.60 ± 0.40
*F*		0.186	0.553	3.454	1.423	20.542	0.449	8.021
*P*		0.666	0.541	0.045	0.233	1.4E-006	0.503	0.005
Maonan	1234							
Carrier	148	5.46 ± 1.27	1.39 (0.60)	2.47 ± 0.43	2.87 ± 0.96	1.23 ± 0.23	0.91 ± 0.21	1.44 ± 0.44
Non-carrier	1086	5.20 ± 1.00	1.53 (0.60)	2.50 ± 0.39	2.84 ± 0.77	1.28 ± 0.15	0.88 ± 0.19	1.55 ± 0.41
*F*		15.074	1.484	3.499	2.333	24.174	2.195	1.164
*P*		1.8E-005	0.266	0.038	0.092	1.8E-007	0.139	0.281

*TC*, total cholesterol; *TG*, triglyceride; *HDL-C*, high-density lipoprotein cholesterol; *LDL-C*, low-density lipoprotein cholesterol; *ApoA1*, apolipoprotein A1; *ApoB*, apolipoprotein B; *ApoA1/ApoB*, the ratio of apolipoprotein A1 to apolipoprotein B. The value of triglyceride was presented as median (interquartile range)for not meet the normal distribution, the difference among the genotypes was determined by the Wilcoxon-Mann-Whitney test. n = sample size.

### Correlated factors for serum lipid parameters

Multivariable linear regression analyses showed that the levels of HDL-C and ApoA1 were associated with the rs2483058 genotypes and/or alleles in Han. The levels of TC were correlated with the rs2580520 genotypes and alleles, HDL-C and ApoA1 with the rs2483058 genotypes in Maonan (Table 8).

**Table 8:** Correlation between serum lipid parameters and the *SRGAP2* alleles/genotypes in the Han and Maonan populations.

Lipid	SNP	Allele	Genotype	Std.error	Beta	*t*	*P*
**Han plus Maonan**							
TC	rs2580520		GG/GC/CC	0.029	0.116	4.531	7.6E-004
HDL-C	rs2483058		GG/GC/CC	0.002	−0.139	−4.903	6.1E-004
	rs2483058	G/C		0.033	0.111	3.978	9.5E-004
ApoA1	rs2483058		GG/GC/CC	0.091	0.091	3.443	0.001
ApoA1/ApoB	rs2483058		GG/GC/CC	0.013	−0.067	−3.048	0.002
	rs2483058	G/C		0.001	−0.071	−2.993	0.003
**Han**							
HDL-C	rs2483058		GG/GC/CC	0.028	−0.081	−2.422	0.016
	rs2483058	G/C		0.054	0.054	2.718	0.044
ApoA1	rs2483058		GG/GC/CC	0.008	−0.136	−4.082	8.1E-004
**Maonan**							
TC	rs2580520		GG/GC/CC	0.056	0.081	2.455	0.014
	rs2580520	G/C		0.095	0.109	2.439	0.015
HDL-C	rs2483058		GG/GC/CC	0.017	−0.074	−2.430	0.015
ApoA1	rs2483058		GG/GC/CC	0.008	−0.103	−3.248	0.001

*TC*, total cholesterol; *HDL-C*, high-density lipoprotein cholesterol; *Apo*, apolipoprotein; *Beta*, standardized coefficient. Association of serum lipid traits and allele and genotypes in Maonan, Han and combined the Maonan and Han populations were assessed by multivariable linear regression analyses with stepwise modeling.

Serum lipid parameters were also correlated with several environmental factors such as sex, age, alcohol consumption, cigarette smoking, blood pressure, blood glucose, waist circumference, and BMI in both ethnic groups or in males and females (*P* < 0.05-*P* < 0.001; Tables 9 and 10).

**Table 9:** Relationship between serum lipid parameters and relative factors in the Han and Maonan populations.

Lipid	Risk factor	B	Std.error	Beta	*t*	*P*
**Han plus Maonan**						
TC	Waist circumference	0.018	0.004	0.147	4.500	0.001
	Age	0.008	0.002	0.101	4.210	9.1E-003
	Ethnic group	0.375	0.043	0.174	8.689	2.1E-008
	Diastolic blood pressure	0.007	0.002	0.070	3.218	0.001
TG	Waist circumference	0.045	0.006	0.228	7.187	4.5E-008
	Age	−0.007	0.003	−0.062	−2.599	0.009
	Alcohol consumption	0.303	0.099	0.073	3.054	0.002
	Body mass index	−0.130	0.053	−0.273	−2.434	0.015
	Glucose	0.099	0.021	0.094	4.740	8.9E-005
HDL-C	Waist circumference	−0.009	0.002	−0.139	−4.903	7.3E-005
	Gender	0.130	0.033	0.111	3.978	7.8E-004
	Cigarette smoking	0.100	0.031	0.074	3.204	0.001
	Age	0.003	0.001	0.077	4.626	9.1E-005
	Alcohol consumption	0.136	0.030	0.095	4.491	9.3E-005
	Ethnic group	−0.520	0.020	−0.452	−25.678	6.4E-011
LDL-C	Ethnic group	−0.073	0.033	−0.045	−2.214	0.027
	Age	0.007	0.001	0.124	5.038	6.4E-005
	Alcohol consumption	−0.120	0.049	−0.060	−2.455	0.014
	Waist circumference	0.015	0.003	0.157	4.771	8.6E-005
ApoA1	Cigarette smoking	0.037	0.091	0.091	3.443	0.001
	Age	0.001	0.001	0.061	2.517	0.012
	Alcohol consumption	0.070	0.010	0.166	6.772	5.4E-006
	Gender	0.039	0.011	0.113	3.524	8.3E-004
	Ethnic group	0.015	0.007	0.146	2.244	0.025
ApoB	Glucose	0.007	0.002	0.061	3.092	0.002
	Age	0.001	0.000	0.085	3.619	7.9E-004
	Ethnic group	0.018	0.008	0.047	2.382	0.017
ApoA1/ApoB	Waist circumference	−0.011	0.002	−0.234	−7.385	3.1E-006
	Age	−0.002	0.001	−0.071	−2.993	0.003
	Gender	0.104	0.006	0.122	3.923	8.4E-004
	Cigarette smoking	0.067	0.025	0.068	2.649	0.008
	Alcohol consumption	0.082	0.025	0.080	3.363	0.001
**Han**						
TC	Waist circumference	0.021	0.007	0.154	3.024	0.003
	Age	0.006	0.003	0.077	2.166	0.030
TG	Waist circumference	0.067	0.011	0.291	5.913	9.1E-006
	Glucose	0.124	0.029	0.122	4.239	9.1E-005
	Cigarette smoking	0.430	0.158	0.104	2.723	0.007
	Systolic blood pressure	0.022	0.005	0.133	4.445	8.8E-005
	Age	−0.015	0.004	−0.117	−3.393	0.001
HDL-C	Gender	0.026	0.057	0.102	2.191	0.029
	Cigarette smoking	0.109	0.054	0.054	2.718	0.044
	Weight	−0.023	0.013	−0.465	−2.406	0.016
	Age	0.006	0.003	0.077	2.166	0.030
	Alcohol consumption	0.135	0.055	0.090	2.460	0.014
LDL-C	Waist circumference	0.011	0.005	0.103	2.022	0.043
	Age	0.006	0.002	0.110	3.069	0.002
	Cigarette smoking	−0.167	0.074	−0.090	−2.257	0.024
ApoA1	Gender	0.001	0.000	0.081	2.294	0.022
	Alcohol consumption	0.087	0.016	0.202	5.563	1.2E-005
ApoB	Glucose	0.008	0.012	0.082	2.455	0.012
	Systolic blood pressure	0.001	0.002	0.233	6.678	6.3E-006
	Age	0.001	0.001	0.079	2.034	0.041
ApoA1/ApoB	Glucose	−0.021	0.007	−0.091	−3.156	0.002
	Gender	0.147	0.019	0.173	3.805	9.3E-004
**Maonan**						
TC	Waist circumference	0.018	0.005	0.155	3.518	9.8E-004
	Gender	0.231	0.095	0.109	2.439	0.015
	Age	0.011	0.003	0.150	4.329	9.2E-005
TG	Waist circumference	0.031	0.007	0.181	4.242	9.5E-005
	Alcohol consumption	0.478	0.123	0.126	3.873	9.3E-004
	Height	−0.089	0.024	−0.467	−3.677	9.6E-004
	Weight	0.131	0.033	0.912	3.968	0.001
	Body mass index	−0.251	0.071	−0.605	−3.550	9.8E-004
HDL-C	Waist circumference	−0.011	0.002	−0.252	−5.933	8.6E-006
	Systolic blood pressure	−0.002	0.001	−0.121	−2.908	0.004
	Diastolic blood pressure	0.004	0.001	0.117	3.004	0.003
	Age	0.002	0.001	0.087	2.615	0.009
	Alcohol consumption	0.149	0.031	0.155	4.809	7.6E-005
	Cigarette smoking	0.075	0.030	0.078	2.256	0.024
LDL-C	Age	0.009	0.002	0.157	4.547	8.4E-005
	Waist circumference	0.018	0.004	0.202	4.601	8.5E-005
ApoA1	Alcohol consumption	0.042	0.015	0.103	2.866	0.004
	Age	0.002	0.001	0.087	2.615	0.009
	Gender	0.050	0.015	0.146	3.263	0.001
	Cigarette smoking	0.042	0.015	0.103	2.866	0.004
ApoB	Waist circumference	0.005	0.002	0.291	5.366	5.1E-005
	Age	0.002	0.001	0.233	4.52	8.3E-005
ApoA1/ApoB	Age	−0.004	0.001	−0.118	−3.547	9.8E-004
	Waist circumference	−0.014	0.002	−0.313	−7.406	3.3E-006
	Alcohol consumption	0.113	0.054	0.110	3.417	9.8E-004

*TC*, total cholesterol; *TG*, triglyceride; *HDL-C*, high-density lipoprotein cholesterol; *LDL-C*, low-density lipoprotein cholesterol; *ApoA1*, apolipoprotein A1; *ApoB*, apolipoprotein B; *ApoA1/ApoB*, the ratio of apolipoprotein A1 to apolipoprotein B; *B*, unstandardized coefficient; *Beta*, standardized coefficient. Association of serum lipid traits and environment exposures in Maonan, Han and combined the Maonan and Han populations were assessed by multivariable linear regression analyses with stepwise modeling.

**Table 10:** Relationship between serum lipid parameters and relative factors in the males and females of the Han and Maonan populations.

Lipid	Risk factor	B	Std.error	Beta	*t*	*P*
**Han/male**						
TC	Diastolic blood pressure	0.011	0.004	0.120	2.474	0.014
TG	Glucose	0.161	0.071	0.110	2.260	0.024
	Age	−0.022	0.010	−0.128	−2.220	0.028
	Diastolic blood pressure	0.028	0.012	0.156	2.473	0.014
HDL-C	Weight	−0.043	0.014	0.750	−2.958	0.001
	Glucose	−0.036	0.014	−0.125	2.635	0.009
	Age	0.006	0.002	0.172	3.072	0.002
ApoA1	Age	0.002	0.001	0.129	2.291	0.022
ApoB	Systolic blood pressure	0.003	0.001	0.161	2.764	0.006
	Glucose	0.022	0.007	0.187	3.233	0.001
ApoA1/ApoB	Weight	−0.041	0.017	−0.789	−2.448	0.015
	Alcohol consumption	0.247	0.075	0.178	3.301	0.001
	Glucose	−0.030	0.015	−0.114	−2.011	0.045
**Han/female**						
TC	Age	0.015	0.004	0.178	3.729	9.4E-004
TG	Diastolic blood pressure	0.017	0.004	0.170	4.336	8.8E-005
	Glucose	0.100	0.021	0.170	4.783	6.7E-005
LDL-C	Pulse pressure	0.006	0.002	0.089	2.267	0.024
ApoB	Cigarette smoking	−0.132	0.057	−0.127	−2.677	0.008
	Diastolic blood pressure	0.004	0.001	0.218	4.646	6.9E-005
	Age	0.003	0.001	0.185	3.465	0.001
ApoA1/ApoB	Age	−0.008	0.002	−0.203	−3.752	9.4E-004
	Diastolic blood pressure	−0.005	0.002	−0.114	−2.393	0.017
	Cigarette smoking	0.521	0.140	0.179	3.723	9.4E-004
**Maonan/male**						
TC	Glucose	0.132	0.020	0.187	4.361	8.8E-005
TG	Alcohol consumption	0.530	0.193	0.120	2.745	0.006
HDL-C	Waist circumference	−0.019	0.003	−0.403	−5.417	5.5E-005
	Alcohol consumption	0.182	0.035	0.219	5.188	5.6E-005
LDL-C	Alcohol consumption	0.259	0.073	0.161	3.570	9.8E-004
	Age	0.007	0.03	0.129	2.292	0.022
ApoA1	Cigarette smoking	0.040	0.017	0.101	2.333	0.020
	Alcohol consumption	0.069	0.018	0.174	3.939	9.6E-004
	Diastolic blood pressure	0.003	0.001	0.152	2.569	0.011
ApoB	Age	0.003	0.001	0.199	3.193	0.002
	Glucose	0.016	0.008	0.107	2.106	0.036
ApoA1/ApoB	Age	−0.006	0.003	−0.127	−1.977	0.049
	Alcohol consumption	0.247	0.075	0.178	3.301	0.001
**Maonan/female**						
TC	Glucose	−0.097	0.029	−0.128	−3.400	0.001
	Age	0.013	0.003	0.168	3.838	9.5E-004
TG	Pulse pressure	0.005	0.002	0.115	2.907	0.004
	Waist circumference	0.029	0.004	0.314	6.503	3.3E-004
HDL-C	Waist circumference	−0.007	0.002	−0.153	−3.065	0.002
	Glucose	−0.027	0.010	−0.103	−2.771	0.006
LDL-C	Alcohol consumption	0.551	0.167	0.117	3.308	0.001
ApoB	Age	0.003	0.001	0.186	3.650	9.7E-004
	Pulse pressure	0.001	0.001	0.118	2.517	0.012
ApoA1/ApoB	Pulse pressure	−0.003	0.001	−0.138	−2.922	0.004
	Age	−0.004	0.002	−0.143	−2.778	0.006

*TC*, total cholesterol; *TG*, triglyceride; *HDL-C*, high-density lipoprotein cholesterol; *LDL-C*, low-density lipoprotein cholesterol; *ApoA1*, apolipoprotein A1; *ApoB*, apolipoprotein B; *ApoA1/ApoB*, the ratio of apolipoprotein A1 to apolipoprotein B; *B*, unstandardized coefficient; *Beta*, standardized coefficient. Association of serum lipid traits and environment exposures in males and females of the both ethnic groups were assessed by multivariable linear regression analyses with stepwise modeling.

### Relative factors for serum lipid phenotypes

**Figure 4:**
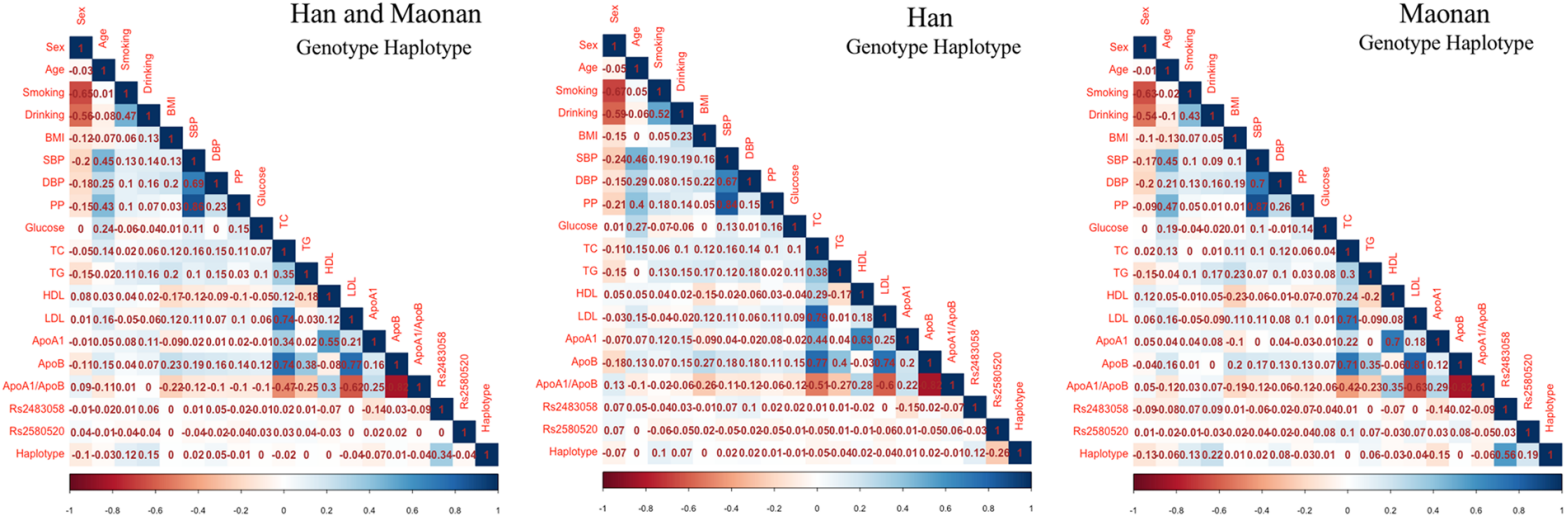
Correlation between environmental exposures and serum lipid variables, as well as the candidate loci.

As shown in Fig. 4, Pearson correlation analysis demonstrated that the integrative variants and haplotype connected with the *SRGAP2* rs2483058 and rs2580520 SNPs to lipid variables. Several environmental exposures such as age, gender, cigarette smoking, alcohol consumption and traditional cardiovascular risk factors such as BMI and blood pressure levels were also correlated with serum lipid phenotypes in the both ethnic groups.

## Discussion

The present study demonstrated that different *SRGAP2* SNPs had different effect on serum lipid traits that (i) the levels of HDL-C, ApoA1 and the ratio of ApoA1 to ApoB in Maonan were different among the rs2483058 genotypes; (ii) the levels of TC in Maonan males and females and the ratio of ApoA1 to ApoB in Han and the level of LDL-C and ApoB in Maonan were different among the three rs2580520 genotypes; (iii) the levels of HDL-C and ApoA1 in males or females were different in Maonan and Han in rs2483058 genotypes; and (iv) the levels of TC and the ratio of ApoA1 to ApoB in males or females were different in Maonan and Han in rs2580520 genotypes.

In the current study, we found that the rs2483058C allele carriers had lower ApoA1 levels and the ApoA1/ApoB ratio than the rs2483058C allele non-carriers in both ethnic groups. The rs2483058C allele carriers also had lower HDL-C concentration than the rs2483058C allele non-carriers in Maonan but not in Han. The rs2580520C allele carriers had higher TC, LDL-C and ApoB levels than the rs2580520C allele non-carriers in Maonan but not in Han. The levels of TG, LDL-C and ApoB in the two ethnic groups were not significantly different among the rs2483058 and rs2580520 genotypes. It is well-known that dyslipidemia is a complex disease caused by environmental and genetic factors. Previous family and twin studies have suggested that in numerous populations, about 40-60% of the variation in serum lipid profiles is genetically determined^32–36^.

In some previous studies, there were associations with intra-pair differences in HDL-C, rs2483058 in an intron of *SRGAP2*, where twins carrying the C allele were more sensitive to environmental factors^37^. In the present study, we showed that the genotypic frequencies of our populations were similar to those obtained in other populations and to data available in the International HapMap Project’s database (http://www.hapmap.org) for the populations. The frequency of CC, GC and GG genotypes was 42.9%, 42.9% and 14.2% in Utah residents with ancestry from Northern and Western Europe (CEU); 7.2%, 56.4% and 36.4% in Yoruba in Ibadan, Nigeria (YRI); 9.3%, 37.2% and 53.4% in Japanese in Tokyo, Japan (JPT) and 9.5%, 40.5% and 50% in Han Chinese in Beijing, China (HCB). The frequency of C and G alleles was 64.3% and 35.7% in CEU; 27.9% and 72.1% in YRI; 35.5% and 64.5% in JPT and 29.8% and 70.2% in HCB. The present study identified significant differences in the genotypic frequency of the rs2483058 SNP between the two ethnic groups. The frequency of the CC genotype was higher in the Maonan than in the Han population and the frequency of the C allele was higher in the Maonan than in the Han population (41.86% *vs*. 37.03%; *P* = 0.001). These results suggest that the prevalence of the *SRGAP2* rs2483058 SNP may exhibit a racial/ethnic difference.

In some previous studies, Jiang *et al*. had found that the rs2580520 genotypes in Chinese women may have different breast cancer susceptibility, which may contribute to the development of breast cancer in this population^27^. Coleman *et al*. also showed that obesity has been attributed as a risk factor for breast cancer development for over five decades and the contribution of obesity to postmenopausal breast cancer risk is thought to be related to elevated level of circulating estrogens in these women, resulting from aromatase-mediated conversion of androgens to estrogens in peripheral adipose tissue (and possible dysregulation of aromatase expression) and lower level of sex hormone-binding globin^38^. It is well-known that cholesterol is an important material for the synthesis of estrogen and higher cholesterol can give rise to more estrogen synthetic that may be enhanced a possibility to be suffered from breast cancer^39, 40^. The results of the present study clearly showed that the levels of TC were higher in who carried rs2580520C allele than without carried rs2580520C allele.

Important intra- and inter-genetic LD associations have been found in this study. These LD patterns in *SRGAP2* were rather weak and low specific to the population under study and indicated the functional dependencies of the encoded proteins. In the present study, haplotype analysis with the two SNPs further supported the association between *SRGAP2* polymorphisms and serum lipid levels in our study populations. The haplotype of rs2483058G-rs2580520C was the commonest one and represented about 45% of the samples. The haplotypes of rs2483058C-rs2580520C, rs2483058G-rs2580520G and rs2483058C-rs2580520G were associated with increased risk of dyslipidemia, but only rs2483058C-rs2580520G haplotype with a significant meaning. In addition, carriers of rs2483058C-rs2580520G haplotype had decreased serum concentration of HDL-C, ApoA1 and the ratio of ApoA1 to ApoB and increased serum concentration of TC. We also found that haplotypes could explain much more serum lipid variation than any single SNP alone.

The environmental factors just as dietary patterns, lifestyle, obesity, physical activity, and hypertension would also be play an important role in modifying serum lipid levels^41–43^. The dietary habits were different between the Han and Maonan populations. Rice is the Maonan people’s staple food supplemented with corn, sweet potato and other grains. Maonan people preferred to eat spicy and acid food with lots of oil and salt. This preference of high in carbohydrates may be related to the higher blood glucose levels, weight, BMI and waist circumference in Maonan than in Han people. In the meantime, rich oil and salt can give rise to higher blood pressure, serum TC, LDL-C and ApoB levels in Maonan than in Han people. There were lots of past studies had proved that diet alone could account for the variability on serum lipid levels^44, 45^. In the present study, we also found that serum lipid parameters were also correlated with several environmental factors such as age, alcohol consumption, cigarette smoking, blood pressure, blood glucose, waist circumference, and BMI. It is commonly accepted that the high-fat diet especially containing large quantities of saturated fatty acids, raise serum cholesterol concentrations and predispose subjects to coronary artery disease^46^. We also showed that the percentages of individuals who consumed alcohol were higher in Maonan than in Han. Although the effects of alcohol intake on LDL-C appear to vary by specific patient types or patterns of alcohol intake, and perhaps by population and sex hormone, this topic has been the focus of much recent research^47^. A recent study in older Italian individuals (65–84 years old) has found that alcohol intake reached higher serum LDL-C levels^48^. Another recent study of Turks also found increased LDL-C, ApoB and TG with alcohol in men, whereas decreased TG and did not change LDL-C or ApoB with alcohol in women^49^. Therefore, the results of exposure to different lifestyle and environmental factors probably further modify the association of genetic variations and serum lipid levels in our study populations.

The present study has some shortcomings. At first, the size of our study populations is a bit small, which might not have the power to detect the LD across the *SRGAP2* locus. Next, the levels of body weight, waist circumference and the percentages of subjects who smoked cigarettes or consumed alcohol were higher in Maonan than in Han. Although age, BMI, blood pressure, cigarette smoking, and alcohol consumption have been adjusted for the statistical analysis, we cannot completely exclude the influence of these factors on serum lipid levels among different genotypes in both nationalities. In addition, because we selected the SNPs from literature and did not cover the extensive *SRGAP2* locus, we might miss some information from other SNPs.

In summary, the genotypic and allelic frequencies of the *SRGAP2* rs2483058 and rs2580520 SNPs were different between Maonan and Han. There were four haplotypes identified in our study populations. The *SRGAP2* SNPs and rs2483058C-rs2580520G haplotype were closely sexually dimorphic associated with serum lipid traits. The haplotypes can explain much more serum lipid variation than any single SNP alone, especially for serum TC, HDL-C and ApoA1 levels.

## Materials and Methods

### Subjects

The study populations including 1210 unrelated subjects (465 males, 38.43% and 745 females, 61.57%) of Han and 1234 unrelated participants (500 males, 40.52% and 734 females, 59.48%) of Maonan were randomly selected from our previous stratified randomized samples. The participants were all agricultural workers from Huanjiang County, Guangxi Zhuang Autonomous region, People’s Republic of China. The participants’ age ranged from 25 to 80 years with a mean age of 55.77 ± 13.89 years in Han and 56.31 ± 13.80 years in Maonan; respectively. The age distribution and gender ratio were matched between the two groups. All participants were essentially healthy with no history of CVD such as coronary artery disease, stroke, diabetes, hyper- or hypo-thyroids, and chronic renal disease. They were free from medications known to affect serum lipid levels. The investigations were carried out following the rules of the Declaration of Helsinki of 1975 (http://www.wma.net/en/30publications/10policies/b3/), revised in 2008. The Ethics Committee of the First Affiliated Hospital, Guangxi Medical University approved the study protocol (No: Lunshen-2014-KY-Guoji-001; Mar. 7, 2014) prior to data collection, and all participants provided informed consent by signature or by fingerprint (if the participant was illiterate) after they had been informed of the objectives, benefits, medical items and confidentiality agreement of personal information. All procedures and methods were performed in accordance with the relevant ethical guidelines.

### Epidemiological survey

The epidemiological survey was carried out using internationally standardized methods, following a common protocol^50^. Information on demographics, socioeconomic status, and lifestyle factors was collected with standardized questionnaires. Alcohol consumption was categorized into groups of grams of alcohol per day: 0 (non-drinker), <25 and ≥25. Smoking status was categorized into groups of cigarettes per day: 0 (non-smoker), <20 and ≥20. Several parameters such as blood pressure, height, weight, waist circumference, and BMI were measured. The methods of measuring above parameters were referred to previous studies^51^.

### Biochemical analyses

A fasting venous blood sample of 5 ml was drawn from the participants. A part of the sample (2 mL) was collected into glass tubes and used to determine serum lipid levels. Another part of the sample (3 mL) was transferred to tubes with anticoagulants (4.80 g/L citric acid, 14.70 g/L glucose and 13.20 g/L tri-sodium citrate) and used to extract deoxyribonucleic acid (DNA). Measurements of serum TC, TG, HDL-C, and LDL-C levels in the samples were performed by enzymatic methods with commercially available kits (RANDOX Laboratories Ltd., Ardmore, Diamond Road, CrumlinCo. Antrim, United Kingdom, BT29 4QY; Daiichi Pure Chemicals Co., Ltd., Tokyo, Japan). Serum ApoA1 and ApoB levels were detected by the immunoturbidimetric immunoassay using a commercial kit (RANDOX Laboratories Ltd.). All determinations were performed with an auto-analyzer (Type 7170 A; Hitachi Ltd., Tokyo, Japan) in the Clinical Science Experiment Center of the First Affiliated Hospital, Guangxi Medical University^52, 53^.

### DNA amplification and genotyping

Genomic DNA of the samples was isolated from peripheral blood leucocytes according to the phenol-chloroform method^52, 53^. The extracted DNA was stored at 4 °C until analysis. Genotyping of the *SRGAP2* rs2483058 and rs2580520 SNPs were performed by polymerase chain reaction and restriction fragment length polymorphism (PCR-RFLP). PCR amplification was performed using rs2483058: 5′-CAGGGGGCACAGATAGTGGA-3′ as the forward and 5′-GCCAAGTTTGACTACGTGGG-3′ and rs2580520: 5′-AACAGGTTGGGGTGAGCATA-3′ as the forward and 5′-CCCCATCAGTACATCGTGGT-3′ as reversed primer pair (Sangon, Shanghai, People’s Republic of China), respectively. Each 25 μL PCR reaction mixture consisted of 2.0 μL genomic DNA, 1.0 μL each primer (10 μmol/L), 12.5 μL of 2 × *Taq* PCR Master mix (constituent: 0.1 U *Taq* polymerase/μL, 500 μM dNTP each and PCR buffer.), and 8.5 μL of ddH_2_O (DNase/RNase-free). PCR was performed with an initialization step of 95 °C for 5 min, followed by 30 s denaturing at 95 °C, 30 s of annealing at 60 °C and 35 s of elongation at 72 °C for 32 cycles. The amplification was completed by a final extension at 72 °C for 7 min. Following electrophoresis on a 2.0% agarose gel with 0.5 µg/mL ethidium bromide, the amplification products were visualized under ultraviolet light. Subsequently, each restriction enzyme reaction was performed with 5.0 μL amplified DNA, 8.8 μL nuclease-free water, 1.0 μL of 10 × buffer solution and 0.2 μL *Ava*I for rs2483058 and *HPY188*I for rs2580520 restriction enzyme in a total volume of 15 µL digested at 37 °C overnight. After restriction enzyme digestion of the amplified DNA, genotypes were identified by electrophoresis on 2% ethidium-bromide stained agarose gels and visualized with UV illumination. Genotypes were scored by an experienced reader blinded to the epidemiological and serum lipid results. Both of six samples (GG, GC and CC genotypes in two; respectively) detected by the PCR-RFLP were also confirmed by direct sequencing with an ABI Prism 3100 (Applied Biosystems) in Shanghai Sangon Biological Engineering Technology & Services Co., Ltd., People’s Republic of China.

### Diagnostic criteria

The normal values of serum TC, TG, HDL-C, LDL-C, ApoA1, ApoB levels and the ApoA1/ApoB ratio in our Clinical Science Experiment Center were 3.10–5.17, 0.56–1.70, 1.16–1.42, 2.70–3.10 mmol/L, 1.20–1.60, 0.80–1.05 g/L and 1.00–2.50, respectively. The individuals with TC > 5.17 mmol/L and/or TG > 1.70 mmol/L were defined as dyslipidemia^54^. Hypertension was diagnosed according to the 1999 and 2003 criteria of the World Health Organization-International Society of Hypertension Guidelines for the management of hypertension^55, 56^. The diagnostic criteria of overweight and obesity were according to the Cooperative Meta-analysis Group of China Obesity Task Force. Normal weight, overweight and obesity were defined as a BMI < 24, 24–28 and > 28 kg/m^2^, respectively^57^.

### Statistical analyses

The statistical analyses were performed with the statistical software package SPSS 22.0 (SPSS Inc., Chicago, Illinois). The quantitative variables were presented as mean ± standard deviation (serum TG levels were presented as medians and interquartile ranges for not a normal distribution). Allele frequency was determined via direct counting, and the Hardy-Weinberg equilibrium was verified with the standard goodness-of-fit test. The genotype distribution between the two groups was analyzed by the chi-square test. General characteristics between two ethnic groups were compared by the Student’s unpaired *t*-test. The association between genotypes and serum lipid parameters was tested by covariance analysis (ANCOVA). Any SNPs associated with serum lipid profiles at the value of *P* < 0.025 (corresponding to *P* < 0.05 after adjusting for 2 independent tests by the Bonferroni correction) were considered statistically significant. Gender, age, BMI, blood pressure, alcohol consumption and cigarette smoking were adjusted for the statistical analysis. Haploview (Broad Institute of MIT and Harvard, USA, version 4.2) analyzed the haplotype frequencies and pair-wise LD among the detected SNPs. Unconditional logistic regression was used to assess the correlation between the risk of hyperlipidemia and genotypes. Multivariable linear regression analyses with stepwise modeling were used to determine the correlation between the genotypes (GG = 1, GC = 2, CC = 3) and several environmental factors with serum lipid levels in males and females of Han and Maonan populations. Two sides *P* value < 0.05 was considered statistically significant. The heart-map of inter-locus models was measured by R software (version 3.3.0)^58^.
